# How Much of the Variation in the Mutation Rate Along the Human Genome Can Be Explained?

**DOI:** 10.1534/g3.114.012849

**Published:** 2014-07-03

**Authors:** Adam Eyre-Walker, Ying Chen Eyre-Walker

**Affiliations:** School of Life Sciences, University of Sussex, Brighton, BN1 9QG, United Kingdom

**Keywords:** *de novo* mutation, human mutation, cryptic variation, mutation rate variation, mutation rate

## Abstract

It has been claimed recently that it may be possible to predict the rate of *de novo* mutation of each site in the human genome with a high degree of accuracy [Michaelson *et al.* (2012), Cell 151: 1431−1442]. We show that this claim is unwarranted. By considering the correlation between the rate of *de novo* mutation and the predictions from the model of Michaelson *et al.*, we show there could be substantial unexplained variance in the mutation rate. We investigate whether the model of Michaelson *et al.* captures variation at the single nucleotide level that is not due to simple context. We show that the model captures a substantial fraction of this variation at CpG dinucleotides but fails to explain much of the variation at non-CpG sites.

It has been known for some time, from comparative studies, that the mutation rate varies at a number of different scales along the human genome, from variation between individual nucleotides, to differences between whole chromosomes (Hodgkinson and Eyre-Walker 2011). Much of this variation has remained unexplained (Hodgkinson and Eyre-Walker 2011). However, Michaelson *et al.* (2012) recently have claimed that the rate of mutation at each site is highly predictable. They use principle component logistic regression fitted to a dataset of 653 *de novo* mutations (DNMs) to estimate a model from which they can predict the mutability index (MI), a measure of the mutation rate, of each site in the human genome. To assess the fit of the model, they count the number of sites in the genome with a particular MI (*n*) and the number of DNMs at those sites (*d*). They therefore have a prediction of the mutation rate from their model, the MI, and the observed rate of mutation, *z*=*d*/*n*. They find a very strong correlation between the logarithm of *z* and MI and infer that

MI was highly predictive of site-specific (1 bp resolution) mutation rates … and could explain 90% of the variability in mutation rates at sites across the genome…..We conclude that our statistical model of mutability can explain a majority of the variance in site-specific mutation rates.

However, for each MI value they have thousands to millions of sites. As a consequence, any variation that their model does not explain will be averaged out when they consider the observed number of mutations. This can be illustrated as follows. Consider sites with an MI such that their mutation rate is 10^−8^, approximately the mean mutation rate in humans (1000 Genomes Project Consortium 2010; Awadalla *et al.* 2010; Conrad *et al.* 2011). If the model of Michaelson *et al.* (2012) explains all the variation in the mutation rate then all sites with this MI will have a mutation rate of 10^−8^. However, if there is unexplained variance, the mutation rate of each site will deviate from this value. Let us assume that equal numbers of sites with this MI actually have mutation rates of 0.1 × 10^−8^ and 1.9 × 10^−8^; the mean mutation rate for sites with this MI is still 10^−8^. It is clear that if we only sample a few sites, then the observed mean mutation rate often will deviate substantially from the average value of 10^−8^, and the correlation between the log of the observed number of DNMs and the MI will be correspondingly weak if we have similar levels of variation at sites with other MI values. However, as we sample more and more sites, the mean value will approach the expected value of 10^−8^ and the correlation between the log of the number of DNMs per site and MI will become better [assuming that the model of Michaelson *et al.* (2012) explains at least some of the variance]. Because there are typically thousands if not millions of sites for each MI value, any unexplained variance will be averaged away.

Here we examine how much variation there could be that is unexplained by the model of Michaelson *et al.* (2012). We also test whether their model explains a source of variation in the mutation rate that is both strong and likely to be difficult to explain – cryptic variation in the mutation rate. This is variation that exists at the single nucleotide level but which appears to be independent of the context of the adjacent nucleotides. Cryptic variation was first discovered in mammalian nuclear DNA by noting that there is an excess of single-nucleotide polymorphisms (SNPs) that occur at the same site in humans and chimpanzees, even when the influence of the adjacent nucleotides on the mutation rate is taken into account (Hodgkinson *et al.* 2009; see also Leffler *et al.* 2013). A similar excess has been noted for single-nucleotide substitutions in pairs of independent primate species (Johnson and Hellmann 2011). This excess of SNPs does not appear to be due to ancestral polymorphism, sequence assembly errors, or balanced polymorphism because the allele frequencies at sites with SNPs in both humans and chimpanzees are no different to other polymorphic sites (Johnson and Hellmann 2011; see also additional discussion in Johnson and Hellmann 2011 and at http://www.plosbiology.org/annotation/listThread.action?root=21243). Estimates suggest there might be as much variation in the mutation rate that is not associated with context as there is associated with context (Hodgkinson *et al.* 2009). However, predicting whether a site is cryptically hyper- or hypomutable is likely to be difficult, because the increase or decrease in the mutation rate is extremely precise—it affects one nucleotide and not the adjoining sites—and it does not depend on the adjacent nucleotides.

## Materials and Methods

### Data

We downloaded details of DNMs from the supplementary information of several publications (Conrad *et al.* 2011; Iossifov *et al.* 2012; Kong *et al.* 2012; Michaelson *et al.* 2012; Neale *et al.* 2012; O’Roak *et al.* 2011; Sanders *et al.* 2012). If necessary these were lifted over to hg18, the human genome assembly used by Michaelson *et al.* (2012).

### Simulating data

We simulated data under the model of Michaelson *et al.* (2012) as follows. First, for a dataset of DNMs we regressed, using weighted regression, the log of the observed number of DNMs per site, *z*, against MI, to yield the relationship between the mutation rate and MI under the Michaelson model. Because there are a limited number of DNMs for some MI values, we binned the MI values into groups of 10 and removed those bins that had 5 or fewer DNMs. Using the regression equation, and the number of sites, we predicted the expected number of mutations at sites with an MI of *x*, Z(*x*). To generate data under the assumption that the Michaelson model explains all the variance in the mutation rate, we sampled from a Poisson distribution with expected values *Z*(*x*). To investigate the effect of variance unexplained by the Michaelson model, we added an additional step to the simulation. Having used the regression model (of log(DNMs per site) *vs.* MI) to predict the expected number of mutations for a site with an MI of *x*, *Z(x)*, we multiplied this by a random variate drawn from a lognormal distribution with variance = *ϒ/n*, where *n* is the number of sites and ϒ is the variance in the mutation rate at each site unexplained by the Michaelson model. This yielded the expected number of mutations for sites with an MI of *x*; to generate the observed number we sampled from a Poisson distribution. The logic is as follows; the mean mutation rate for sites with an MI of *x* is *Z(x)*, but the rate of a particular site is *Z(x)α* where α is a random variate that is lognormally distributed. Because the mean of *n* lognormally distributed variates, each with a variance *ϒ*, is itself approximately lognormal with a variance equal to *ϒ/n* (Beaulieu *et al.* 1995; Fenton 1960), we can simulate the effect of unexplained variation among sites with an MI of *x* by multiplying the expected mutation rate by a random lognormal variate with variance *ϒ/n*. We generated 1000 simulated datasets and calculated the correlation between MI and the log of the simulated number of mutations per site. Occasionally the simulation would generate no DNMs for an MI value; we removed these datasets. We then compared the correlation between the log of the observed number of DNMs and MI against the correlation between the log of the simulated number of DNMs and MI. To take into account the uncertainty in the relationship between the log of the observed mutation rate and MI, we bootstrapped the data before performing the regression by resampling the data points from the regression.

### Coincident SNP analysis

We investigated the difference in the mutation rate between sites with and without a coincident SNP (a site with a SNP in both humans and chimpanzees) as follows. We assume that the distribution of mutation rates is a gamma distribution arbitrarily scaled such that the mean of the distribution is one; it is therefore characterized solely by its shape parameter. We also assume that hypermutable sites destroy themselves when they mutate. This assumption makes little difference to the non-CpG analysis but reduces the level of variation needed to explain the coincident SNPs in the CpG analysis. Hodgkinson *et al.* (2009) have shown that under this model the probability of observing a coincident SNP at a site is

$$P=u_{h}u_{c}\int D\left(\gamma \right)\left(e^{-v\gamma }\gamma^{2}+\left(1-e^{-2v\gamma }\right)\right)d\gamma$$ (1)

where *u_h_* and *u_c_* are the density of SNPs in the two species being considered, *v* is the average divergence between the species and *D(γ)* is the distribution of the rates. Therefore the average mutation rate of sites with coincident SNPs, relative to the average mutation rate (arbitrarily set to one) is

$$Q=\frac{\int \left(D\left(\gamma \right)e^{-v\gamma }\gamma^{2}+\left(1-e^{-2v\gamma }\right)\right)\gamma d\gamma }{\int \left(D\left(\gamma \right)e^{-v\gamma }\gamma^{2}+\left(1-e^{-2v\gamma }\right)\right)d\gamma }$$ (2)

In our calculations, we assume that the divergence at non-CpG sites between human and chimpanzee sites is 0.0092 (Chimpanzee-Sequencing-and-Analysis-Consortium 2005) with the divergence at CpG sites 10x higher at 0.092 (Chimpanzee-Sequencing-and-Analysis-Consortium 2005; Hwang and Green 2004).

We used the coincident SNPs found in humans and chimpanzees discovered by Leffler *et al.* (2013). We did not exclude coincident SNPs that they had inferred to be subject to balancing selection because there were <300 of these of a total of 34,000 coincident SNPs

## Results and Discussion

Michaelson *et al.* (2012) assessed the fit of their model by comparing the mutation rate predicted by their model to the observed number of DNMs. In doing so, they averaged the mutation rate across thousands if not millions of sites, potentially hiding unexplained variance in the mutation rate. To investigate how much unexplained variance there might be, we simulated data under their model (henceforth referred to as the Michaelson model) with and without additional variance. In the simulation we estimated the relationship between MI (*i.e.*, the Michaelson model) and the rate of mutation using sets of DNMs. We then used this relationship to predict the expected number of mutations at a site and then simulated data based on these expectations with and without additional variation in the mutation rate (details in the section *Materials and Methods*). We performed the analysis for three sets of DNMs: (i) the 652 DNMs reported by Michaelson *et al.* (2012) and used to build the model upon which the MI values are based (referred to as the Michaelson data), (ii) 1380 DNMs reported by various other studies (Conrad *et al.* 2011; Iossifov *et al.* 2012; Neale *et al.* 2012; O’Roak *et al.* 2011; Sanders *et al.* 2012) (Other data), and (iii) 4933 DNMs reported by Kong *et al.* (2012) (Kong data; note that only DNMs with an MI value were included).

**Figure 1:**
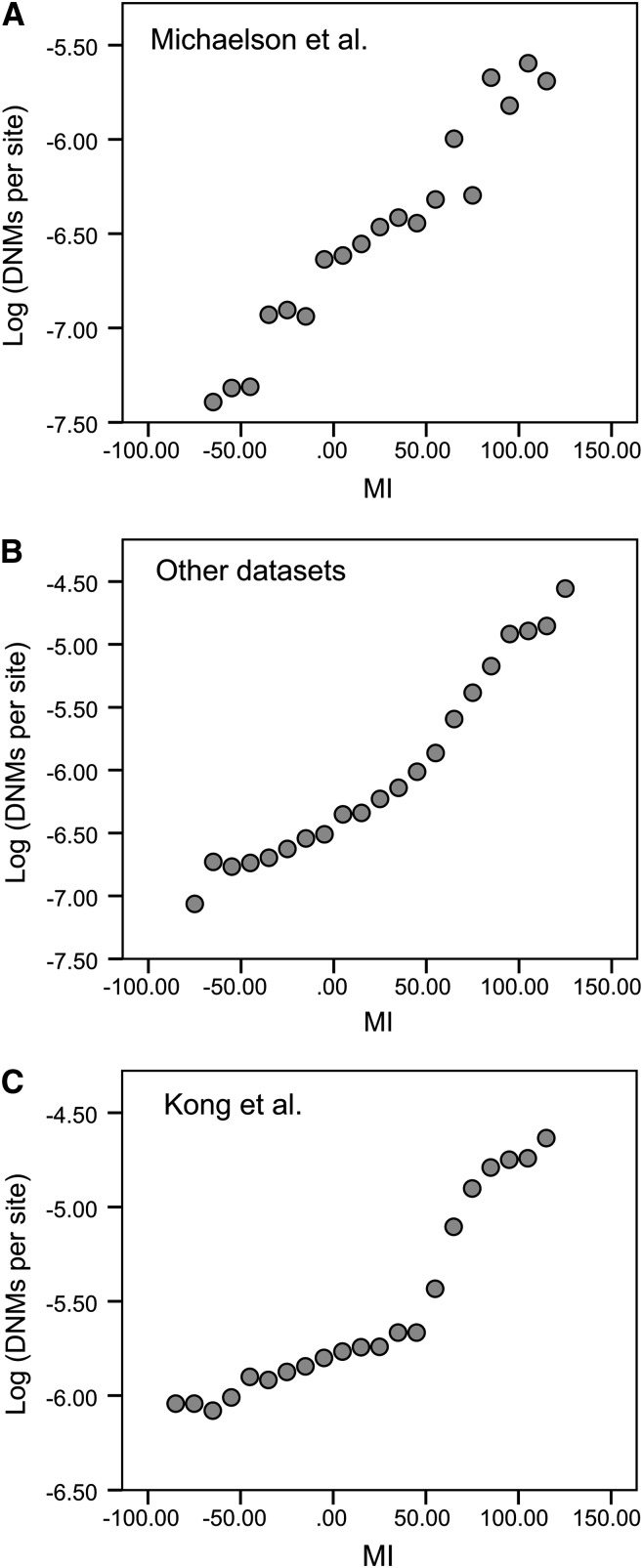
The log of the number of *de novo* mutations (DNMs) per site *vs.* the mutability index (MI). The mutability index for the (A) Michaelson, (B) Other, and (C) Kong datasets.

As previously shown by Michaelson *et al.* (2012), the correlation between the log of the number of DNMs per site and the MI value is very strong for the Michaelson data (r = 0.98, *P* < 0.001; Figure 1A); this finding is perhaps not surprising, given that these were the data used to construct the Michaelson model and the model is relatively parameter rich. However, as Michaelson *et al.* (2012) showed, their model also fits the data from other studies well (r = 0.97, *P* < 0.001; Figure 1B), although there is a clear nonlinearity in the relationship (a quadratic term in a nonlinear regression is significant *P* = 0.010). However, the fit of the Michaelson model to the Kong data, which Michaelson *et al.* (2012) did not study, is relatively poor (r = 0.94, *P* < 0.001; Figure 1C). The problem would seem to lie with the Kong data, since the model fits the other two datasets well. The slope of the regression line from the Kong data [0.0047 (0.0006)] is significantly less than that observed for the Michaelson *et al.* (2012) [0.010 (0.0007)] and Other datasets [0.0084 (0.0007)], suggesting that there has been systematic underreporting of DNMs from the more mutable areas of the genome in the Kong *et al.* (2012) dataset (or alternatively, that there are large numbers of false positives in the less mutable parts).

If we assume that the Michaelson model explains all the variation in the mutation rate, we find that simulated datasets have similar levels of correlation between the log of the number of DNMs per site and MI to that observed in the real data for the Michaelson and other datasets, *i.e.*, any variance not explained by the Michaelson model can be attributed to sampling error due to the fact that there are limited number of DNMs (Table 1). All the simulated correlations are stronger than the observed correlation in the Kong data, but this is probably because the Michaelson model fits these data poorly.

**Table 1:** The proportion of simulated datasets with a greater correlation between the log of the number of DNMs per site and MI than observed in the actual data

*v*	Michaelson *et al.* (2012)	Other	Kong *et al.* (2012)
0	0.81	0.78	1.0
1000	0.82	0.79	1.0
10,000	0.81	0.76	1.0
100,000	0.76	0.72	0.99
500,000	0.59	0.49	0.92
1,000,000	0.43	0.35	0.70
2,000,000	0.25	0.17	0.36
3,000,000	0.16	0.088	0.20
4,000,000	0.10	0.069	0.13
5,000,000	0.076	0.032	0.084

DNMs, *de novo* mutations; MI, mutability index.

However, despite the good fit between model and data for two of the datasets, we find that there could be very substantial levels of unexplained variance and the correlations would remain almost unaffected (Table 1). Only when the variance associated with the unexplained variance approaches 10^5^ do we see the correlations being affected and approaching the values seen in the real data. This level of variance dwarfs that explained by the Michaelson model; the coefficient of variation in the mutation rate explained by the Michaelson model is 1.10, the coefficient of variation for the unexplained variation is 300 if the variance is 10^5^. This analysis therefore shows that there could be a substantial amount of unexplained variance that would never be detected, assessing model fit as Michaelson *et al.* (2012) have done. However, it does not show that this unexplained variance exists.

Assessing model fit is not easy with these datasets; there are very few DNMs spread across millions of sites. We therefore sought to test one component of mutation rate variation that is both substantial and likely to be difficult to predict, the so-called cryptic variation in the mutation rate (Hodgkinson *et al.* 2009; Johnson and Hellmann 2011). This is variation at the single-nucleotide level that is independent of adjacent nucleotides.

To investigate whether the model of Michaelson *et al.* (2012) captures cryptic variation in the mutation rate we proceeded as follows. Sites with SNPs in both humans and chimpanzees are inferred to have greater mutation rates than sites without coincident SNPs. Using some simple theory, we can estimate how much more mutable these sites need to be on average to yield the observed excess of coincident SNPs, and we can then compare this with the difference in mutation rate predicted by the Michaelson model for sites with and without coincident SNPs. If the Michaelson model captures cryptic variation in the mutation rate then it should correctly predict the level of variation needed to explain the observed excess of coincident SNPs.

Although coincident SNPs and DNMs are measuring variation in the mutation rate over very different time scales, it is expected that any source of variation identifiable through coincident SNPs should also be present in DNMs. In fact if variation in the mutation rate evolves through time, we would expect the variation associated with coincident SNPs to be less than that present in DNMs.

Leffler *et al.* (2013) have shown, using a carefully curated dataset of human and chimpanzee SNPs, that there is a 16% excess of coincident SNPs at CpG sites (95% confidence intervals of 14% and 17%) and a 83% (80%, 86%) excess at non-CpG sites between human and chimpanzee, when correcting for the nucleotides at the adjacent sites. Assuming that rates are gamma distributed across sites, we estimate, using the theory set out by Hodgkinson *et al.* (2009), that sites with coincident SNPs are 1.40-fold (95% confidence intervals of 1.35, 1.43) and 2.71-fold (2.65, 2.77) more mutable on average than the genomic average for CpG and non-CpG sites respectively. How do these values compare to those under the Michaelson model? Under the Michaelson model, we find that sites with coincident SNPs have significantly greater MI values at both CpG (mean MI for coincident sites = 91.6, noncoincident sites = 81.4; *P* < 0.001) and non-CpG sites (coincident sites = −7.77, noncoincident sites = −16.0, *P* < 0.001). However, the differences in MI are small and equate to minor differences in the mutation rate predicted using the regression model from the data of Michaelson *et al.* (2012); coincident SNPs are predicted to be 1.21-fold (1.21, 1.21) more mutable at CpG and 1.17-fold (1.17, 1.17) more mutable at non-CpG sites. Thus, our analysis suggests that the Michaelson model may capture a substantial fraction, although by no means all, of the variation at CpG sites; the level of variation required to explain the excess of coincident SNPs at CpG sites is such that we would expect sites with coincident SNPs to be 1.40-fold (1.35, 1.43) more mutable than noncoincident sites and the Michaelson model predicts them to 1.21-fold (1.21, 1.21) more mutable; these estimates are significantly different (*P* < 0.001). However, the Michaelson model seems to fail to capture most of the variation at non-CpG sites; sites with coincident SNPs are expected to be 2.71-fold (2.65, 2.77) more mutable than noncoincident sites, but the Michaelson model predicts them to be only 1.17-fold (1.17, 1.17) more mutable. These estimates are significantly different (*P* < 0.001).

Although the Michaelson model appears to explain a substantial fraction of the variation at CpG sites this might be deceptive because the level of cryptic variation may have been underestimated by analyzing coincident SNPs. Cryptic variation is, by its definition, variation that does not depend upon the context of the immediately adjacent nucleotides. However, it is possible, in fact likely, that cryptic variation depends upon context in some manner, may be through a dispersed context (for example, if the second nucleotide upstream is an A and fifth nucleotide downstream is a C, etc). If this is the case then as the context evolves so the mutation rate at the focal site will change. This will lead to a reduction in the correlation in the mutation rate between sites in humans and chimpanzees and hence to a lower level of variation inferred from coincident SNPs. However, one might argue that the level of variation is overestimated by a consideration of coincident SNPs, because SNPs can be subject to natural selection, which will tend to increase the number of coincident SNPs by excluding SNPs from certain parts of the genome. This seems unlikely because less than 10% of the human genome is inferred to be subject to selection (Eory *et al.* 2010; Lunter *et al.* 2006).

Currently we do not have a robust estimate of how much of the total variance in the mutation rate is contributed by cryptic variation. Initial rough estimates suggest that there is approximately as much variation associated with cryptic variation as there is with simple context (*i.e.*, CpG type effects) (Hodgkinson *et al.* 2009). Because variation at the single-nucleotide level appears to contribute the most to variation in the mutation rate (Hodgkinson and Eyre-Walker 2011) it would seem that the Michaelson model is missing a major component of the variation, particularly at non-CpG sites.
